# Antibiotic resistance pattern of the allochthonous bacteria isolated from commercially available spices

**DOI:** 10.1002/fsn3.2433

**Published:** 2021-06-29

**Authors:** Éva György, Éva Laslo, Márta Antal, Csaba Dezső András

**Affiliations:** ^1^ Department of Food Science Faculty of Economics, Socio‐Human Sciences and Engineering Sapientia Hungarian University of Transylvania Miercurea Ciuc Romania; ^2^ Department of Bioengineering Faculty of Economics, Socio‐Human Sciences and Engineering Sapientia Hungarian University of Transylvania Miercurea Ciuc Romania

**Keywords:** antibiotic resistance, *Bacillus* spp., food, pathogenic bacteria, spices, spoilage bacteria

## Abstract

Spices are often used in dried form, sometimes with significant microbial contamination including pathogenic and food spoilage bacteria. The antibiotic resistance represents an additional risk for food industry, and it is worthy of special attention as spices are important food additives. During our work, we examined the microbiological quality of 50 different spices with cultivation methods on diverse selective media. The identification of the most representative bacteria was carried out using 16S rDNA gene sequence analysis. Antibiotic resistance profiling of twelve identified *Bacillus* species (*B. subtilis subsp. stercoris* BCFK, *B. licheniformis* BCLS, *B. siamensis* SZBC, *B. zhangzhouensis* BCTA, *B. altitudinis* SALKÖ, *B. velezensis* CVBC, *B. cereus* SALÖB isolate, *B. tequilensis* KOPS, *B. filamentosus* BMBC, *B. subtilis subsp. subtilis* PRBC2, *B. safensis* BMPS, and *B. mojavensis* BCFK2 isolate) was performed using the standard disk‐diffusion method against 32 antibiotics. The study showed that the majority resistance was obtained against penicillin G (100%), oxacillin (91.67%), amoxyclav (91.67%), rifampicin (75%), and azithromycin (75%). Our findings suggest that spices harbor multidrug‐resistant bacteria.

## Graphical abstract

During our work, we examined the microbiological quality of 50 different spices with cultivation methods on diverse selective media. The identification of the most representative bacteria was carried out using 16S rDNA gene sequence analysis. Antibiotic resistance profiling of twelve identified *Bacillus* species was performed using the standard disk‐diffusion method against 32 antibiotics. Our findings suggest that spices harbor multidrug‐resistant bacteria

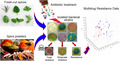

## 1 INTRODUCTION

Spices are various plant parts that contain flavor and aroma compounds that in small amounts enhance the enjoyment value of foods. Nowadays, spices play a key role in modern food preparation as they contribute to salt reduction, reduce the utilization of artificial additives, impart natural color to foods, and act as natural antioxidants (Clemenson, 2019). Spices have been found to provide health benefits based on their bioactive ingredients. Diverse spices and herbs, namely anise, cinnamon, black cumin, curry, coriander, ginger, fenugreek, turmeric, garlic, mustard, pepper, and onion, are potentially involved in diabetes control (Sanlier & Gencerb, 2020).

Spices are additives used in small quantities, owing to a high potential for microbial contamination as they are used in many food commodities. This possibility may be attributed to the existing various critical points (vulnerabilities) of microbial contamination during plant cultivation and product supply chain (Alegbeleye et al., 2018; Székács et al., 2018). Even though the spices have low water activity, they could be harbored by diverse pathogenic and spoilage microorganisms (Costa et al., 2020; Melo González et al., 2017). As dried herb products, they may be contaminated by different molds too.

Due to the high tolerance to dehydration stress, *Salmonella* species can survive longer in dry products such as spices. The contamination can occur at several phases of the production including cultivation, harvesting, processing, storage, packaging, and distribution (Zweifel & Stephan, 2012). Different spices and spiced food caused *Salmonella* outbreaks (Sagoo et al., 2009).

*Bacillus* species are among the most usually detected bacteria species from diverse spices samples with different geographical origin (Antai, 1988; Banerjee & Sarkar, 2003; Chakraborty et al., 2020; Hariram & Labbé, 2015). Contamination of spices with molds and spore‐forming *B. cereus* is attributed to environmental conditions of manufacturing and distribution (Fogele et al., 2018). The survival of these microorganisms in spices and herbs is attributed to the high resistance of spores to different stress conditions including excessive pH values, heat, and low water activity (Frentzel et al., 2018). The result of improper food handling is that *B. cereus*, originating from herbs and spices, can reach the 10^5^–10^6^ CFU/g causing food poisoning (Sagoo et al., 2009). Some strains are capable of producing nonhemolytic enterotoxin, hemolysin BL, and cytotoxin K responsible for diarrhea and emesis causing heat stable cereulide (Frentzel et al., 2018).

Among the spore‐forming bacteria also, *Bacillus subtilis* and *Clostridium perfringens* are often present (Sagoo et al., 2009). Other bacteria strains like S*taphylococcus aureus, Escherichia coli,* and *Shigella* spp. were detected in different spices (Banerjee & Sarkar, 2003). In contrast to *Bacillus* spores, *S*. *aureus* has a short life cycle even though it supported by the low water activity conditions (Thanh et al., 2018).

In dried vegetables and spices, certain lactic acid bacteria were found, such as *Enterococcu*s spp., *Leuconostoc* spp., *Lactobacillus* spp., *Weissella confusa*, *W. cibaria, W. paramesenteroides, Pediococcus acidilactici,* and *P. pentosaceus* (Säde et al., 2016).

The herbs in many cases are used as a fresh cut product without any or with improper treatment to combat microbial contamination. The contaminated plant material can take part in the transmission of pathogens to foods that provide suitable growth conditions and toxin production (Thanh et al., 2018). This increases the health hazard to consumers. The risk is increased in the case of multidrug‐resistant strains present on the plant surface.

The overuse of antibiotics selected the antimicrobial resistance of bacteria, and it represents a virulence factor resulting in a worldwide health threat to humans. Bacterial strains with drug resistance are listed as causes of human mortality (Bennani et al., 2020; Thapa et al., 2020). Bacterial strains with antibiotic resistance can enter the food supply chain from different environments (Dutta & Ramamurthy, 2020; Thapa et al., 2020). Diverse food commodities may act as a reservoir and transmission vector of antibiotic resistance due to contamination by bacteria (Mercimek Takci et al., 2020; Navaneethan & Effarizah, 2021). In food producing animals, bacterial strains like *Campylobacter jejuni* and *Campylobacter coli* resistant to quinolones and fluoroquinolones were determined. It was shown that *E. coli, Salmonella* spp., and *C. jejuni* harbor resistance to tetracyclines. Another association was found between macrolides and *E. coli, Salmonella enterica serovar Heidelberg,* and *C. coli* (Bennani et al., 2020).

The spread of antibiotic resistance genes is an increasing problem, and it is also indicated that clinically relevant resistance could be detected in nonpathogenic bacterial strains as in the case of different *Bacillus* spp. strains originating from natural ecosystems (Berić et al., 2018). The occurrence of the *B. cereus* group as opportunistic pathogen has been associated with vegetables. These bacterial groups display resistance to commonly used antibiotics. Multidrug‐resistant strains were also identified among them. These bacteria can contribute to the transfer of antibiotic resistance genes in the food chain (Fiedler et al., 2019; Navaneethan & Effarizah, 2021). According to Zarzecka et al., (2020), there is a risk of transmission of antibiotic resistance genes to pathogenic bacteria like *S. aureus, B. cereus* and others.

The utilization of antimicrobials exceeding the therapeutic use in food production and agriculture may promote the transmission of antibiotic genes (Wang et al., 2019). To prevent the dissemination of antibiotic resistance, the knowledge and estimation of allochthonous bacteria and their antibiotic pattern are necessary.

This study aimed to evaluate the microbiological quality of different spices and to determine the antibiotic resistance of the most representative bacterial isolates originating from the studied spice. With this study, we attempt to find an answer to the question of whether spices could represent a reservoir of multidrug‐resistant strains and whether their spread in the food supply chain is possible.

## 2 MATERIALS AND METHODS

### 2.1 Isolation of bacterial strains

The microbial contamination of 50 different commercially available spices (allspice, Cayenne pepper, marjoram, coriander, basil, cinnamon, granulated garlic, ground garlic, bay leaf, ground pepper, Provence spice mix, rosemary, chili peppers, ginger, star anise, savory, oregano, caraway seeds, ground cumin, tarragon, pepper mix, white mustard, parsley leaf, ground hot peppers, juniper berries, ground turmeric, sweet peppers, cloves, lovage, nutmeg, ground sage, crushed sage, ground vanilla, spice mixture, pepper+garlic mixture, curry, ground anise, green pepper, peppermint, cardamom, mustard seeds, ground cloves, dried celery, dill, parsley) was determined with cultivation methods on different selective media. During the microbiological assay, the total mesophilic aerobic bacteria on Nutrient agar (Himedia) medium were first determined. The incubation was performed at 37℃ for 48 hr. Thereafter, we determined the presence of aerobic spore‐forming *B. cereus, E. coli*, *Salmonella* spp., and *Pseudomonas* species on selective agar mediums. The detection of *C. perfringens* was carried out in Clostridial Differential Broth (Biolab). In the case of a positive result, a confirmation test (with regenerated milk) was conducted and the enumeration of *C. perfringens* was determined with the most probable number method. In 5 test tubes containing 10 ml of double concentrated Clostridial Differential Broth, it was added 10 ml of stock suspension from the samples. In 5 test tubes containing 10 ml of Clostridial Differential Broth, it was added 1 ml of stock suspension. 1–1 ml from 10^–1^ serial dilutions was transferred in 5 test tubes containing 10 ml of standard *Clostridium* selective broth. In the inoculated tubes, it was added paraffin and incubated for 10 min at 80℃ in water bath. After cooling, it was incubated for 48 hr at 44℃. The positive tubes were counted, and the number of *Clostridium perfringens* was determined from the MPN table (Drăgan‐Bularda, 2000). For the detection and enumeration of aerobic spore‐forming *B. cereus,* we used ChromoBio® Cereus Base Agar (Biolab). For the detection and enumeration of *E. coli,* TBX Chromo Agar (Oxoid) was used. Brilliance ^TM^ Salmonella Agar Base (Oxoid) was used for the detection of *Salmonella* species and Pseudomonas Isolation Agar Base (Himedia) for the detection and enumeration of *Pseudomonas* spp. The incubation was performed at 37℃ for 48 hr. Bacterial colonies with high number and characteristic colony morphology were isolated, and pure cultures were made.

### 2.2 Identification of the isolated bacterial strains

The identification of these bacteria was done using 16S rDNA gene sequence analysis. Genomic DNA isolation was carried out with AccuPrep^®^ Genomic DNA Extraction Kit from Bioneer according to the manufacturer's protocol. An universal primer set 27f and 1492r (5’ AGAGTTTGATCMTGGCTCAG 3’, 5’ TACGGYTACCTTGTTACGACTT 3’) was used to amplify one part of the bacterial 16S rDNA gene. The amplification reaction was carried out with an initial denaturation at 94℃ for 5 min, followed by 30 cycles consisting of denaturation at 94℃ for 30 s, primer annealing at 55℃ for 30 s, and primer extension at 72℃ for 1 min, and a final extension at 72℃ for 7 min. Sequencing of the amplified PCR products of the isolated strains was performed by commercial service of Biomi KFT (Hungary) (György et al., 2020). The resulted sequences were edited and aligned with Chromas (Technelysium Pty. Ltd., South Brisbane, Australia); Molecular Evolutionary Genetics Analysis 4 system (www.megasoftware.net) was used forphylogenetic analyses. The isolated bacteria were identified through comparison of the sequences using the EzTaxon server based on the 16SrDNA sequence data (www.ezbiocloud.net/eztaxon).

### 2.3 Determination of antibiotic resistance

The next part of the research was the determination of antimicrobial resistance of isolated and identified 12 bacterial species belonging to the *Bacillus* genus (*B. cereus* SALÖB isolate, *B. licheniformis* BCLS, *B. altitudinis* SALKÖ, *B. safensis* BMPS, *B. filamentosus* BMBC, *B. zhangzhouensis* BCTA, *B. velezensis* CVBC, *B. tequilensis* KOPS, *B*. *siamensis* SZBC, *B. mojavensis* BCFK2 isolate, *B. subtilis subsp*. *subtilis* PRBC2, and *B. subtilis subsp*. *stercoris* BCFK) from various spices. The antibiotic susceptibility testing was performed by the disk diffusion method according to European Committee on Antimicrobial Susceptibility Testing (EUCAST) guidelines.

A total of 32 different antibiotic disks containing the antibiotics nalidixic acid 30 µg (NA), imipenem 10 µg (IPM), meropenem 10 µg (MRP), rifampicin 5 µg (RIF), tigecycline 15 µg (TGC), erythromycin 15 µg (E), colistin (methane sulfonate) 10 µg (CL), levofloxacin 5 µg (LE), amikacin 30 µg (AK), linezolid 30 µg (LZ), kanamycin 30 µg (K), cefotaxime 30 µg (CTX), ofloxacin 5 µg (OF), clindamycin 2 µg (CD), ceftazidime 30 µg (CAZ), ceftriaxone 30 µg (CTR), teicoplanin 30 µg (TEI), tobramycin 10 µg (TOB), cefoperazone 75 µg (CPZ), azithromycin 15 µg (AZM), oxocillin 1 µg (OX), amoxyclav (amoxicillin/clavulonic acid) 30 µg (AMC), ciprofloxacin 5 µg (CIP), trimethoprim 5 µg (TR), streptomycin 10 µg (S), cefoxitin 30 µg (CX), ampicillin 10 µg (AMP), vancomycin 30 µg (VA), tetracycline 30 µg (TE), gentamicin 10 µg (GEN), penicillin G 10 unite (P), and chloramphenicol 30 µg (C) were used. Onto the surface of Mueller‐Hinton agar medium (Himedia), 0.1ml suspension of bacteria (10^8^ CFU/ml) taken in study was inoculated and the antibiotic disks were placed. This was followed by incubation for 24 hr at 37℃. The diameter (mm) of inhibition zone was measured, and the results of antimicrobial resistance test in accordance with interpretive guidelines and breakpoints were described as susceptible (S), intermediate (I), and resistant (R) (EUCAST, 2016).

### 2.4 Determination of multiple antibiotic resistance index

The multiple antibiotic resistance (MAR) index of the screened bacterial strains was calculated with the help of the following formula: MAR index =x/y, where (x) is the number of antibiotics, to which the bacteria species was resistant, and (y) refers to the number of tested antibiotics defined for multidrug resistance (Costa et al., 2020).

### 2.5 Statistical analysis

The principal component analysis was performed with Statistica 8.0 (StatSoft, Inc., Oklahoma, USA).

## 3 RESULTS

During the evaluation of the microbiological quality of spices, it was determined the aerobic mesophilic bacteria count. If this is high, it can be assumed that pathogenic bacteria may also be present in the samples. The highest total plate count was detected in the case of ground pepper 1⋅5∙10^7^ CFU/g, but a high total colony count was characteristic for turmeric (4⋅10^5^ CFU/g), tarragon (3.8⋅10^4^ CFU/g), and savory1 (1⋅7.10^4^ CFU/g) samples (Table 1). The number of the mesophilic aerobic bacteria was low in green pepper, vanilla, oregano1, spice mixture, and white mustard samples. The plate count of aerobic spore‐forming bacteria was highest in the allspice sample (3.5⋅10^4^ CFU/g), and the lowest count was detected in vanilla and mustard seed samples.

**TABLE 1:** Allochthonous bacteria count in studied spices (CFU/g)

Spice sample and origin	Aerobic mesophilic bacteria	Aerobic spore‐forming bacteria	*Bacillus cereus*	*Escherichia coli*	*Salmonella* spp.	*Pseudomonas* spp.
Allspice (Hungary)	2.8⋅10^4^	3⋅5.10^4^	1.3⋅10^2^	<10	<10	<10
Basil (Egypt)	9.9⋅10^3^	8.7⋅10^3^	2⋅10	<10	<10	<10
Bay leaf (Turkey)	1.4⋅10^2^	7.1⋅10^2^	<10	<10	<10	<10
Caraway seed (Poland)	1⋅10	2⋅10	<10	<10	<10	<10
Cardamom (Austria)	1.6⋅10	6.6⋅10^2^	<10	<10	<10	<10
Cayenne pepper (non‐EU)	3.8⋅10^3^	3.1⋅10^2^	1⋅10	<10	<10	<10
Chili peppers (Poland)	2⋅10	4⋅10	<10	<10	<10	<10
Cinnamon (Indonesia)	5.8⋅10	1.1⋅10^2^	1⋅10	<10	<10	<10
Cloves1 (Zanzibar)	3⋅10	1⋅10	<10	<10	<10	<10
Cloves2 (Zanzibar)	1.1⋅10	8⋅10	<10	<10	<10	<10
Coriander1 (Austria)	5⋅10^2^	4⋅10^3^	<10	<10	<10	<10
Coriander2 (Austria)	5.6⋅10	2⋅10	<10	<10	<10	3⋅2.10^2^
Curry (Poland)	2.4⋅10^2^	2.9⋅10^2^	<10	<10	<10	<10
Dill (Czech Republic)	1⋅10	1.5⋅10^2^	<10	<10	<10	<10
Dried celery (Poland)	2.4⋅10	4⋅10	<10	<10	<10	<10
Ginger (Nigeria)	3.5⋅10	9⋅10	<10	<10	<10	<10
Granulated garlic (Austria)	2.5⋅10	2.1⋅10^2^	<10	<10	<10	<10
Green pepper (Austria)	<10	1.3⋅10^2^	<10	<10	<10	<10
Ground anise (Hungary)	3.3⋅10^4^	2.4⋅10^3^	6⋅10	3.10	7⋅10^2^	<10
Ground cloves (Zanzibar)	3⋅10^2^	7.6⋅10^2^	4⋅10	<10	<10	<10
Ground cumin (Hungary)	4.5⋅10^3^	6.4⋅10^3^	2⋅10	<10	1.1⋅10^2^	<10
Ground garlic (China)	2.7⋅10^2^	2.3⋅10^3^	6⋅10	<10	3⋅10	<10
Ground hot peppers (Brazil)	5⋅10^2^	8.7⋅10^2^	2⋅10	<10	<10	1.10
Ground pepper (Brazil)	1.5⋅10^7^	1⋅10^4^	5⋅10^2^	<10	3⋅10^4^	<10
Juniper berries (Macedonia)	3.5⋅10^2^	3.8⋅10^3^	1⋅10^2^	<10	3⋅10	<10
Lovage (Germany)	1.2⋅10	3⋅10	1⋅10	<10	<10	<10
Marjoram (Germany)	3.8⋅10^2^	1.1⋅10^3^	<10	<10	<10	<10
Pepper mix (Poland)	1⋅10	2⋅10	<10	<10	<10	<10
Mustard seed (Moldova)	<10	<10	<10	<10	<10	<10
Nutmeg (Indonesia)	4⋅10^2^	2⋅10^2^	<10	<10	<10	<10
Oregano1 (Turkey)	<10	1⋅10	<10	<10	<10	<10
Oregano2 (Turkey)	4.3⋅10^4^	1⋅10^2^	<10	<10	4.4⋅10^3^	1.1⋅10^2^
Parsley (Poland)	6.2⋅10^3^	3.1⋅10^3^	5⋅10^2^	<10	7⋅10	<10
Parsley leaf (Poland)	1.4⋅10	4⋅10^2^	<10	<10	<10	<10
Pepper +garlic (Poland)	2.9⋅10	7⋅10^2^	<10	<10	<10	<10
Peppermint (Egypt)	9.4⋅10^3^	1.67⋅10^4^	1.1⋅10^2^	<10	3.2⋅10^3^	6⋅10
Provence spice mix (Austria)	3⋅10^2^	5.6⋅10^3^	<10	<10	<10	<10
Rosemary (Morocco)	3⋅10	3⋅10	<10	<10	<10	<10
Sage1 (Turkey)	1.1⋅10^2^	1.4⋅10^2^	<10	<10	<10	<10
Sage2 (Austria)	3.6⋅10	2.7⋅10^2^	<10	<10	<10	<10
Savory1 (Hungary)	1.7⋅10^4^	1.2⋅10^3^	<10	5⋅10^3^	2.7⋅10^3^	5⋅10^2^
Savory2 (Hungary)	1.4⋅10^3^	8⋅10	<10	<10	<10	<10
Spice mixture (Poland)	<10	1.4⋅10^2^	<10	<10	<10	<10
Star anise (Vietnam)	7.6⋅10^3^	2.4⋅10^3^	<10	5⋅10^3^	<10	<10
Sweet pepper (China)	6.2⋅10^2^	5.3⋅10^2^	7⋅10	5⋅10	9⋅10	<10
Tarragon1 (Poland)	3.8⋅10^4^	6⋅10	<10	3⋅10^3^	5.7⋅10^3^	1.5⋅10^3^
Tarragon2 (Poland)	3.3⋅10^2^	1.8⋅10^2^	<10	<10	<10	<10
Turmeric (India)	4⋅10^5^	2.2⋅10^2^	<10	7⋅10	1.6⋅10^2^	1.4⋅10^2^
Vanilla (Madagascar)	<10	<10	<10	<10	<10	<10
White mustard (Poland)	<10	1⋅10	<10	<10	<10	<10

Within the group of aerobic spore‐forming bacteria, typical *B. cereus* colonies were developed from 15 spice samples, and high count was detected in the case of ground pepper and parsley. *Pseudomonas* species, that play a role in food spoilage, were generally present in small numbers in the assayed spices, with the exception of tarragon1, coriander2, turmeric, savory1, and oregano2 samples. The hygienic indicator bacteria *E. coli* were detected in seven samples, whereas the colony count reached 10^3^ CFU/g in samples star anise, savory1, and tarragon1. The presence of *Salmonella* spp. on the selective medium was found in 12 spice samples, and the highest count was detected in the case of ground pepper. Also, fecal origin *C. perfringens,* anaerobic spore‐forming bacterium, was detected in 7 spice samples: cinnamon, basil, tarragon1, ground pepper, ground cumin, peppermint and sage2. The MPNs of this bacteria were distributed from 5 (in cinnamon) to 1.6⋅10^3^ (in ground cumin and peppermint), respectively, 9 in ground pepper and sage2. In tarragon1, the MPNs were 1.41⋅10^2^ and 5.42⋅10^2^ in basil. Among the examined spices, good microbiological quality with low or no microbiological load could be observed in the case of vanilla, rosemary, chili peppers, ginger, oregano1, cumin seeds, pepper mix, white mustard, cloves1, spice mixture, green pepper, and mustard seed samples.

In the case of some spices (star anise, peppermint, tarragon1, parsley, ground pepper, turmeric, allspice, ground anise), higher numbers of allochthonous bacteria were present, which may result from the poor conditions of cultivation, storage, and distribution.

A total number of 20 bacterial isolates originated from different spices on diverse selective mediums were identified by partial 16S rDNA sequencing. The bacterial isolates were selected based on colony morphology and provenience. The results show (Table 2) that the bacterial isolates belonged to 3 genera: 18 species from *Bacillus* genera, one species from *Staphylococcus,* and one species from *Salinicoccus* spp. Among the identified bacterial species, 15 different species were detected from the dried plant materials.

**TABLE 2:** The source and identification of the bacterial strains isolated from the different spices

Bacterial isolate ID	Source of isolation	Isolation medium	Identified closely related species based on 16S rDNA	Sequence similarity%
BCFK	Spice mix	ChromoBio® Cereus Base	*Bacillus subtilis subsp. stercoris*	98.39
BCLS	Lovage	ChromoBio® Cereus Base	*Bacillus licheniformis*	99.50
ORPS2	Oregano	Pseudomonas Isolation Agar Base	*Bacillus tequilensis*	99.20
KOPS	Coriander	Pseudomonas Isolation Agar Base	*Bacillus tequilensis*	99.90
CUBC	Curry	ChromoBio® Cereus Base	*Bacillus velezensis*	99.30
SALTARK	Tarragon	Brilliance ^TH^ Salmonella Agar Base	*Bacillus zhangzhouensis*	99.30
PRBC2	Parsley leaf	ChromoBio® Cereus Base	*Bacillus subtili*s *subsp*. *subtilis*	96.85
SZBC	Ground cloves	ChromoBio® Cereus Base	*Bacillus siamensis*	97.89
PRBC1	Parsley leaf	ChromoBio® Cereus Base	*Bacillus zhangzhouensis*	98.91
KNA	Dill	Nutrient Agar	*Bacillus zhangzhouensis*	98.01
BCFK2	Spice mix	ChromoBio® Cereus Base	*Bacillus mojavensis*	78.91
BCTA	Tarragon	ChromoBio® Cereus Base	*Bacillus zhangzhouensis*	99.50
BCPR	Sweet peppers	ChromoBio® Cereus Base	*Salinicoccus* spp.	84.62
BMPS	Peppermint	Pseudomonas Isolation Agar Base	*Bacillus safensis*	99.70
BMBC	Peppermint	ChromoBio® Cereus Base	*Bacillus filamentosus*	99.18
ÁNTBX	Ground anise	TBX Chromo agar	*Staphylococcus warneri*	96.00
ÁNSAL	Ground anise	Brilliance ^TH^ Salmonella Agar Base	*Bacillus altitudinis*	99.13
BCKÖ	Ground cumin	ChromoBio® Cereus Base	*Bacillus siamensis*	99.90
SALÖB	Ground pepper	Brilliance ^TH^ Salmonella Agar Base	*Bacillus cereus*	80.85
SALKÖ	Ground cumin	Brilliance ^TH^ Salmonella Agar Base	*Bacillus altitudinis*	100

Because the similarity percentage in the case of SALÖB isolate (*B. cereus* identity 80.85%) and BCFK2 isolate (*B. mojavensis* 78.91%) is very low, these bacteria are marked with isolate designation.

The principal component analysis (PCA) was carried out on the measured values (inhibition zone diameters). PCA was applied to classify the identified bacterial strains based on the inhibition of the different antibiotics. The results of multivariate data analysis give some indication of the complete data set, showing the variability among the bacterial strains regarding antibiotic pattern. The principal components, F1 accounts for 55.10% cumulative variability, F2 for 14.83% from total variability, explained 69.93% of total variability which was high enough to represent all the variables.

**FIGURE 1:**
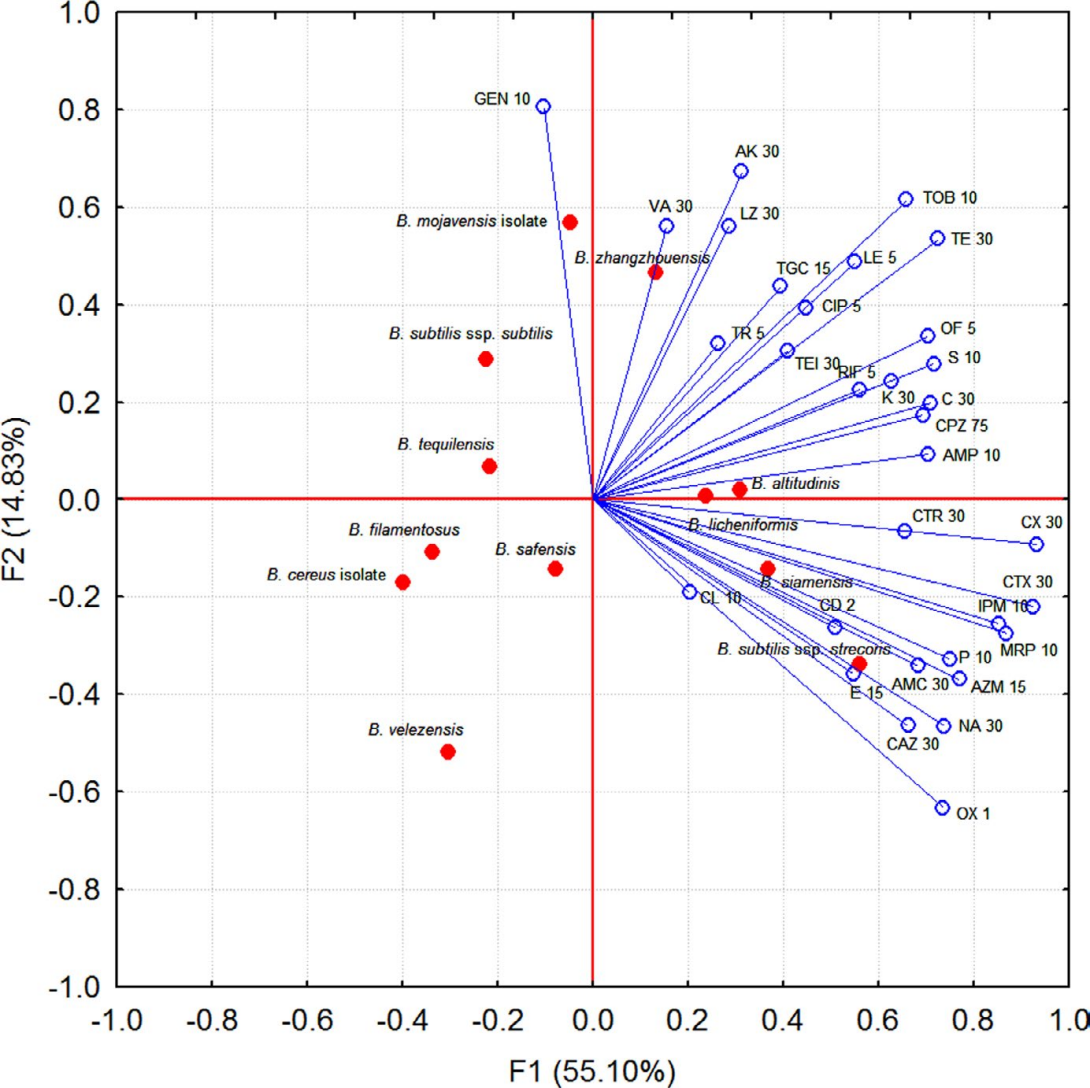
PCA analysis of antibiotic inhibition zones means of the bacterial strains isolated from spices

Half of the bacterial strains were clustered in the upper part of PC2 axis with high PC2 values in F1‐F2 biplot (Figure 1). *B. tequilensis*, *B. subtili*s *subsp*. *subtilis,* and *B. mojavensis* were situated in the upper left quadrant. 12 antibiotics did not affect the growth of these isolates. *B. zhangzhouensis* and *B. altitudinis* were clustered in the upper right zone, were characterized by almost the same mean of inhibition zone, except for penicillin G, imipenem, meropenem, streptomycin, erythromycin, amoxyclav, and oxacillin. *B. filamentosus, B. cereus* isolate*, B. safensis,* and *B. velezensis* were clustered on the left with low negative PC1 values. These bacteria were characterized by 0 mm zone size diameter or low values in the case of the majority of antibiotics. *B*. *lichenformis*, *B*. *siamensis,* and *B*. *subtilis* *subsp. stercoris* were in the lower right quadrant. All three displayed susceptibility to the same twenty antibiotics.

Because of the lack of EUCAST guidelines to determine resistance of *Bacillus* strains, the criteria for the Gram‐positive *Staphylococcus* group were used in our study. According to the results of antibiotic resistance, all the tested *Bacillus* species strains were generally resistant to penicillin G (100%), amoxyclav (91.67%), and oxacillin (91.7%). The majority of the strains (66.67%) showed resistance to clindamycin, imipenem, and meropenem. More than half of the tested bacterial strains displayed resistance to erythromycin, cephotaxime, cefoxitin, and nalidixic acid. Half of the strains exhibited resistance to ceftazidime, streptomycin, and cefoperazone. Kanamycin and chloramphenicol inhibited the growth of four bacterial strains, while ofloxacin inhibited the growth of one bacterial strain. All tested bacterial strains were highly sensitive (100%) to eight antibiotics: ciprofloxacin, linezolid, vancomycin, amikacin, trimethoprim, gentamicin, teicoplanin, tigecycline, and colistin (92.7%) (Table 3).

**TABLE 3:** Antibiotic resistance pattern of bacterial strains isolated from spices

Bacterial strain	Antibiotic resistance
*Bacillus subtilis subsp. stercoris BCFK*	P, CD
*Bacillus licheniformis BCLS*	P, CPZ, CD, C, AMC,OX,CL
*Bacillus siamensis SZBC*	P, RIF, AMC, OX
*Bacillus zhangzhouensis BCTA*	P, RIF,AZM, IPM, MRP,CTX, AMC, OX, NA
*Bacillus altitudinis SALKÖ*	P, RIF, AZM, S, E, AMC, OX
*Bacillus velezensis CVBC*	CX, P, CPZ, RIF, AZM, LE, IPM, MRP, S, C, TE, CTX, AMC, OF, OX, CTR, TOB, AMP
*Bacillus cereus SALÖB*	CX, P, CPZ, RIF, AZM, LE,CAZ, CD, IPM, MRP, S, C, E, CTX, AMC, K, OF, OX, TOB, AMP, NA
*Bacillus tequilensis KOPS*	CX, P, RIF, AZM, CAZ, CD, IPM, MRP, E, CTX, AMC, OF, OX, CTR, AMP, NA
*Bacillus filamentosus BMBC*	CX, P, CPZ, RIF, AZM, CAZ, CD, IPM, MRP, S, C, E, CTX, AMC, K, OX, TOB, NA
*Bacillus subtilis subsp. subtilis PRBC2*	CX, P, RIF, AZM, CAZ, CD, IPM, MRP, S, E, CTX, AMC,K, OX, AMP, NA
*Bacillus safensis BMPS*	CX, P, RIF, AZM, CAZ, CD, IPM, MRP, E, AMC,K, OX, TOB, NA
*Bacillus mojavensis BCFK2*	CX, P, AZM, CAZ, CD, IPM, MRP, S, E, CTX, AMC, OX, NA

Note

CX cefoxitin, P penicillin G, CPZ cefoperazone, RIF rifampicin, AZM azithromycin, LE levofloxacin, LZ linezolid, CAZ ceftazidime, CD clindamycin, IPM imipenem, MRP meropenem, S streptomycin, C chloramphenicol, TE tetracycline, E erythromycin, CTX cephotaxime, AMC amoxyclav, OF ofloxacin, OX oxacillin, CTR ceftriaxone, TOB tobramycin, AMP ampicillin, NA nalidixic acid, TEI teicoplanin, CL colistin.

Four bacterial strains were generally susceptible (33.3%) to seven antibiotics: cefoxitin, cefoperazone, imipenem, meropenem, erythromycin, cephotaxime, and nalidixic acid. Susceptible strains were also determined for levofloxacin (58.3%), ceftazidime (41.7%), chloramphenicol (66.7%), kanamycin (66.7%), ceftriaxone (41.7%), tobramycin (66.7%), and ampicillin (66.7%).

Strains with intermediate susceptibility were also determined for cefoxitin (8.33%), cefoperazone (16.7%), levofloxacin (25%), ceftazidime (8.33%), clindamycin (8.33%), streptomycin (25%), tetracycline (16.7%), erythromycin (8.33%), cephotaxime (8.33%), ceftriaxone (41.7%), and nalidixic acid (8.33%).

The multiple antibiotic resistance (MAR) index was taken into account (the antimicrobial categories and agents) used to define multidrug resistance (due to the lack of data for *Bacillus* strains regarding the antibiotics used to define MAR we applied the antibiotics proposed for *S. aureus)* (Magiorakos et al., 2012).

**FIGURE 2:**
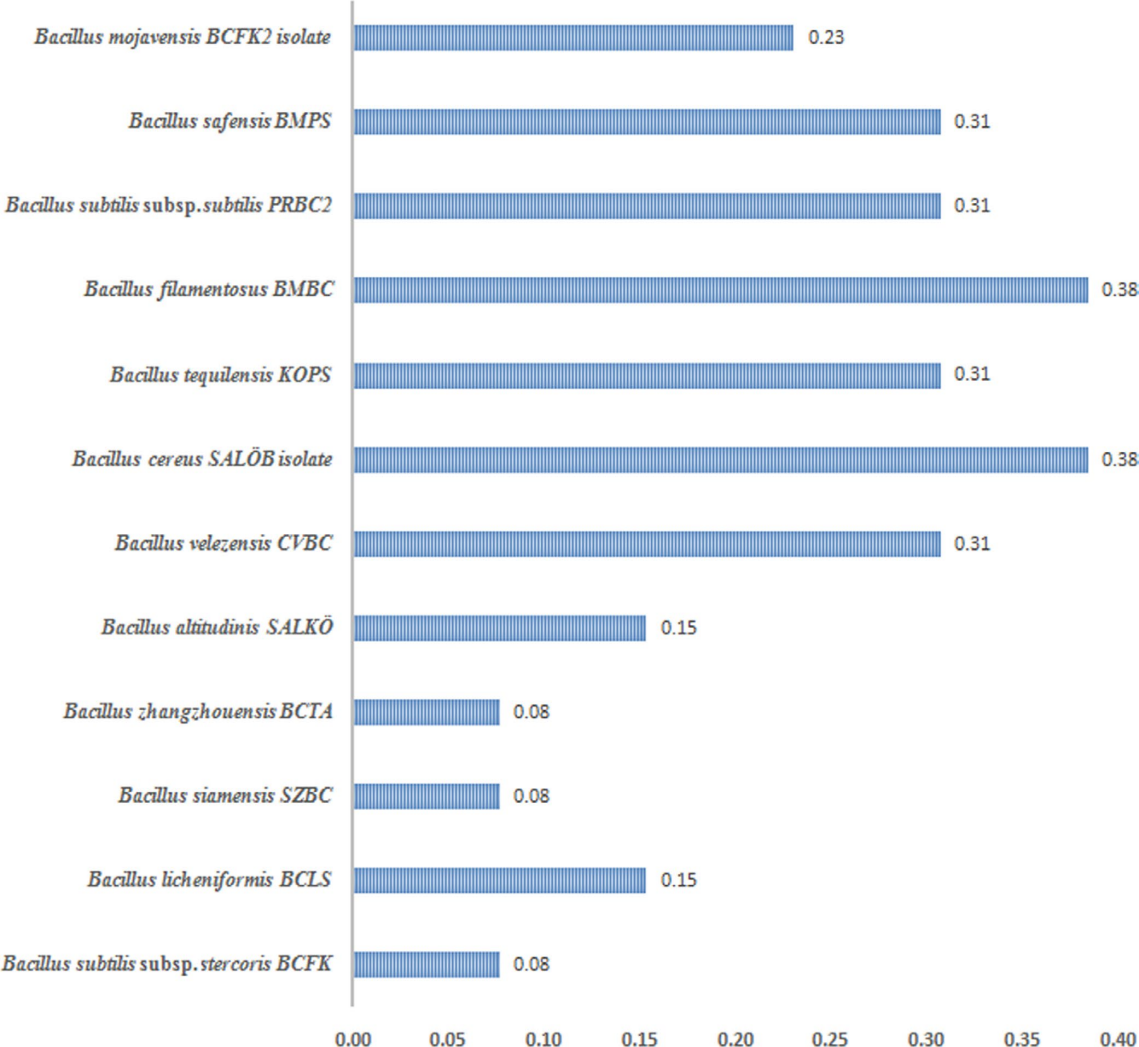
Multiple antibiotic resistance (MAR) index values of *Bacillus* species isolated from spices

When a bacteria strain displays resistance to more than two antibiotics or to at least one antibiotic in three or more categories (Table 3), it can be noted as multiple antibiotic resistance (MAR) bacteria. The multiple antibiotic resistance (MAR) index of the twelve isolated and identified *Bacillus* strains is presented in Figure 2. MAR index of seven out of twelve bacterial strains (*B. velezensis* CVBC, *B. cereus* SALÖB isolate*, B. tequilensis* KOPS*, B. filamentosus* BMBC*, B. subtilis subsp. subtilis* PRBC2*, B. safensis* BMPS, and *B. mojavensis* BCFK2 isolate) is equal to or higher than 0.23. MAR index values in the case of five bacteria strains are below 0.2. *B. zhangzhouensis* BCTA, *B. altitudinis* SALKÖ, *B. licheniformis* BCLS, *Bacillus siamensis* SZBC, and *Bacillus subtilis* s*ubsp*. *stercoris* BCFK possess MAR index in the interval 0.08–0.15. Based on these criteria, seven bacteria strains can be defined as multi antibiotic resistant strains. The values of MAR index higher than 0.2 indicate high‐risk sources of contamination.

## 4 DISCUSSION

Isolation and identification of antibiotic resistant bacteria from different ecosystems including food commodities are carried out by direct plating methods and 16S rRNA gene sequence analysis (Leboffe & Pierce, 2011; Poretsky et al., 2014; Sentausa & Fournier, 2013; Wang, Zhou, et al., 2019).

Microbial quality determinations have shown that spices and herbs can reach high microbial counts of up to 10^8^ colony forming units (CFU/g). Among the isolated microorganisms, there are also pathogenic species that can be responsible for diverse foodborne diseases. The most relevant bacteria strains are *B. cereus, C. perfringens,* and *Salmonella* spp. Among the foodborne pathogens were detected *S. aureus* too (Thanh et al., 2018).

The lack of or the low microbial load of different spices could be attributed to the bioactive components with antimicrobial properties (Costa et al., 2020; Thanh et al., 2018). Bata‐Vidács et al., (2018) determining the microbiological quality of seventy‐one spice paprika samples from 10 countries (Hungary, China, Serbia, Spain, India, Bulgaria, Brazil, Peru, Kenya, Thailand, and unknown place) concluded that the dominant microorganisms in spice paprika samples are influenced by different factors such as the climate of cultivation. It was revealed that bacteria species are related to geographical origin. It was shown that *B. mycoides* and *B*. *licheniformis* take part in the characteristic microflora of spice paprika from Central Europe. The presence of *B. safensis* was determined in samples grown in the tropical monsoon climate. In samples from Spain was detected *B. amyloliquefaciens subsp. plantarum*, *B. amyloliquefaciens subsp. amyloliquefaciens*, and *B. mojavensis*. There were reported outbreaks caused by spices contaminated with *B. cereus* and *B. subtilis* (Moore et al., 2019). Some of these strains listed above were detected in our samples too. *Bacillus* strains detected in our samples possess different attributes described as appearing in different environmental conditions. *B. licheniformis* is often involved in food spoilage because of the extremely resistant endospore forming ability, like in milk powder. It was shown that the endospore is highly resistant to different factors like desiccation, heat, and irradiation. This resistance was attributed to the fact that spores germinate at a reduced rate compared to other bacteria belonging to the *B. subtilis* group (Aspholm et al., 2019; Wang et al., 2019). *B. mojavensis* was reported as endophyte bacteria, isolated from maize showing plant growth promoting benefits and antifungal activity.

The spreading of the antibiotic resistant bacteria and genes represents major public health concerns in the different environments of food commodities like fruits, vegetables, and foods from animals. Preventive measure has been proposed, including awareness of antibiotic resistance and monitoring (Mancilla‐Becerra, Lías‐Macías, Ramírez‐Jiménez, & León, 2019; Thapa et al., 2020). Consumption of foods with spices without any heat treatment that can harbor multidrug‐resistant bacteria represent a danger for immune depressed individuals. These bacteria strains may survive gastrointestinal tract complicating the treatment in persons with lowered immune function (Fiedler et al., 2019).

Antibiotic resistance develops when the bacteria with different mechanisms (target changes as modifications of the penicillin binding protein (PBP), enzymatic inhibition, porin mutations, efflux pumps) survive the effect of antibiotics. The antibiotic mechanisms are named: intrinsic resistance, acquired resistance, genetic changes in DNA, and DNA transfer (Breijyeh et al., 2020). It was confirmed that some classes of antibiotic resistance gene can be acquired. Acquired resistance has confirmed to the most of the standard antibiotics and evaluated in different bacteria (Gold & Moellering, 1996; Kapoor et al., 2017; Mancilla‐Becerra et al., 2019). According to WHO priority list of antibiotic resistant bacteria, *S. aureus* possess high priority, but other bacteria strains are also classified as serious (Breijyeh et al., 2020; Kumar et al., 2020).

Limited scientific information is available regarding the antibiotic resistance of *Bacillus* strains and the transfer of antibiotic resistance genes. According to Alanber et al., (2020), *Bacillus* spp. are able to transfer the antibiotic resistance genes. Agersø et al., (2019) suggested that in *B. licheniformis,* putative genes correlated with the resistance of streptomycin, erythromycin, and chloramphenicol are present in the chromosome and are intrinsic, which means that they are not transferable to other bacteria and consequently, will not take part in the pool of resistance genes. According to Muriuki et al., (2020), vended fast food carries antibiotic resistant bacteria as *B. velezensis* isolated from fish, with tetracycline, chloramphenicol, cefotaxime, and nalidixic acid resistance. *B. subtilis* isolated from vegetable salads exhibited resistance to streptomycin, gentamycin, amoxicillin, tetracycline, chloramphenicol, cefotaxime, nalidixic acid, and trimethoprim +sulfamethoxazole. *B. siamensis* B44, originating from pickled vegetables, displayed susceptibility to 14 antibiotics, whereas the other isolates as *Bacillus* spp. strain B51f were resistant to clindamycin, chloramphenicol, penicillin G, and cephalothin (Meidong et al., 2017).

A bacterial isolate originating from a river was identified as *B. altitudinis* with resistance to three groups of antibiotics beta lactams, lincomycin, and polymyxin B (Lobova et al., 2015). A similar pattern of results was also obtained by Alanber et al., (2020). Generally, the isolated *Bacillus* strains from powdered infant milk and from spices (in our study) were susceptible including amikacin, gentamicin, and imipenem. The *B. cereus* displayed resistance to ceftazidime and *B. licheniformis* showed resistance to cefoperazone and *B. subtilis* was susceptible to ceftazidime. The results regarding the enterotoxin producing foodborne pathogen *B. cereus* are in accordance with findings confirmed by Fiedler et al., (2019). This bacteria strain was detected also in dried herbs and spices showing resistance to standard antibiotics including chloramphenicol, cefotaxime, erythromycin, penicillin, and imipenem. Fei et al., (2020) revealed a high *B. cereus* contamination level in powdered foods, where all the isolates displayed ampicillin, oxacillin, and rifampicin resistance, as in this study.

The multidrug resistance of 12 *Bacillus s*trains against 32 antibiotics is impossible to visualize graphically without using dimension reduction statistical methods. For this purpose, PCA is a widely used and important multivariate statistical approach (Jolliffe & Cadima, 2016; Kassambara, 2017) and could be applied in classifying the antibiotic resistance bacteria. The PCA results showed different groupings of the tested bacteria. In some previous studies also, it applied this method for reduction of multivariate data to two or three principal components. Zhang et al., (2013) carried on PCA and cluster analysis in explaining antibiotic resistance indicators in *E. coli* isolated from aquaculture, indicating that MAR index defined detailed the antibiotic resistance profile. Mandal et al., (2020) performed PCA for assessing the resistance profiles and association of heavy metal tolerance and antibiotic resistance of clinically relevant bacteria, grouped into three factors with high total variance.

## 5 CONCLUSION

The study has revealed that the assayed 50 spices are moderately contaminated. It was shown that among the identified bacteria occur pathogenic and food spoilage bacteria, as *B. cereus*.

The isolated and identified *Bacillus* species exhibited different resistance to the tested antibiotics, showing resistance at least against two antimicrobials. All tested strains showed susceptibility to eight antibiotics. The presence of bacteria with multiple antibiotic resistance in the tested products should be considered a health concern. From the results of the present study, it can be concluded that improperly processed or unwashed raw herb spices could contribute to the transfer of multidrug resistance bacteria through the supply chain. Further studies are needed to confirm with accuracy the minimal inhibitory concentration and detection of genetic elements associated with antibiotic resistance in case of bacteria with human health concern.

## CONFLICT OF INTEREST

The authors declare that they do not have any conflict of interest.

## AUTHOR CONTRIBUTIONS

**Éva György:** Conceptualization (equal); Investigation (equal); Methodology (equal); Writing‐original draft (equal); Writing‐review & editing (equal). **Éva Laslo:** Conceptualization (equal); Investigation (equal); Methodology (equal); Writing‐original draft (equal); Writing‐review & editing (equal). **Márta Antal:** Investigation (equal). **András Csaba Dezső:** Data curation (equal); Formal analysis (equal); Visualization (equal).
